# Pneumothorax Ex-vacuo or “trapped lung” in the setting of hepatic hydrothorax

**DOI:** 10.1186/1471-2466-12-78

**Published:** 2012-12-17

**Authors:** Yan S Kim, Irawan Susanto, Catherine A Lazar, Ali Zarrinpar, Patricia Eshaghian, M Iain Smith, Ronald Busuttil, Tisha S Wang

**Affiliations:** 1 Division of Pulmonary and Critical Care Medicine, Department of Medicine, David Geffen School of Medicine at University of California, 10833 Le Conte Ave, Room 37-131 CHS, Los Angeles, CA, 90095, USA; 2 Dumont-UCLA Liver Cancer and Transplant Centers, Pfleger Liver Institute, Department of Surgery, David Geffen School of Medicine at University of California, Los Angeles, California, USA

**Keywords:** Hepatohydrothorax, Trapped lung, Pnumothorax Ex-Vacuo, Pleural effusion

## Abstract

**Background:**

Hepatic hydrothorax is a major pulmonary complication of liver disease occurring in up to 5-10% of patients with cirrhosis.

**Case presentation:**

We report four observations of the development of pneumothorax ex-vacuo or trapped lung in the setting of hepatic hydrothorax. The diagnosis of trapped lung was made based on the presence of a hydropneumothorax after evacuation of a longstanding hepatic hydrothorax with failure of the lung to re-expand after chest tube placement in three of the four cases. Two patients underwent surgical decortication with one subsequent death from post-operative bleeding. The other two patients remarkably had spontaneous improvement of their “trapped lung” without surgical intervention.

**Conclusions:**

While pneumothorax ex-vacuo is a known phenomenon in malignant effusions, to our knowledge, it has never been described in association with hepatic hydrothoraces. The pathophysiology of this phenomenon remains unclear but could be related to chronic inflammation with development of a fibrous layer along the visceral pleura.

## Background

Hepatic hydrothorax is a major pulmonary complication of end stage liver disease (ESLD). The diagnosis of hepatic hydrothorax can be made in patients with cirrhosis and portal hypertension who develop large, usually right sided, pleural effusions in the absence of any primary cardiac, pulmonary, or pleural disease. Dysregulation of sodium and fluid management in patients with cirrhosis leads to the development of ascites. The ascites can then migrate from the abdomen to the chest through microperforations in the diaphragm leading to a pleural effusion that is transudative by Light’s criteria. This passage can be further aided by the negative pressure in the chest generated during inspiration. The prevalence of hepatic hydrothorax in the setting of cirrhosis ranges from 5-10% [1-3]. The most cited complications of hepatic hydrothorax include respiratory compromise and spontaneous bacterial pleuritis [4]. Management of hepatic hydrothorax is generally symptomatic. In mild cases, sodium restriction and diuretics can be used to minimize the size of the effusion, supplemented when necessary by therapeutic thoracenteses. In refractory cases, consideration can be given to transjugular intrahepatic porto-systemic shunt (TIPS). In this series, we report four observations of pneumothorax ex-vacuo or trapped lung associated with hepatic hydrothorax.

Pneumothorax ex-vacuo was first described as the result of gas being drawn into the pleural space due to acute endobronchial obstruction with lobar collapse that is fully reversible once the obstruction is resolved [5]. The definition we have adopted for this case series is the one described in inflammatory effusions – when the lung is unable to re-expand after evacuation of pleural fluid that develops after a remote episode of inflammation in the setting of a thickened visceral pleura [6,7]. To our knowledge, pneumothorax ex-vacuo has never been reported in association with hepatic hydrothoraces.

## Case presentation

### Case 1

A 24-year-old male with hepatitis C contracted at birth developed cirrhosis complicated by variceal bleeding, portal hypertension, ascites, and a persistent right-sided hepatic hydrothorax that required thoracenteses every four to six weeks. The effusions were transudative in nature (fluid to serum total protein ratio 0.4, fluid to serum LDH ratio 0.5) and without evidence of infection, pleural, or parenchymal abnormalities consistent with a hepatic hydrothorax. Given his refractory effusions, an indwelling pleural cavity drainage catheter was placed at an outside hospital 8 months prior to his orthotopic liver transplantation (OLT) for self-drainage of the effusion at home. The pleural catheter was removed 1 month after its placement when he was referred for OLT in order to minimize the chance of infectious complications. He was subsequently managed successfully with diuretics and therapeutic thoracenteses as needed for symptomatic relief. At the time of liver transplantation he had undergone approximately ten thoracenteses.

**Figure 1:**
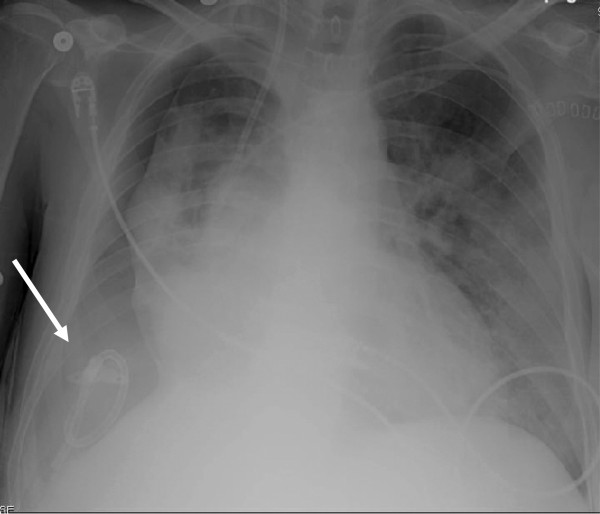
**Chest x-ray post-OLT: Pneumothorax ex-vacuo.** Arrow indicates location of chest tube.

**Figure 2:**
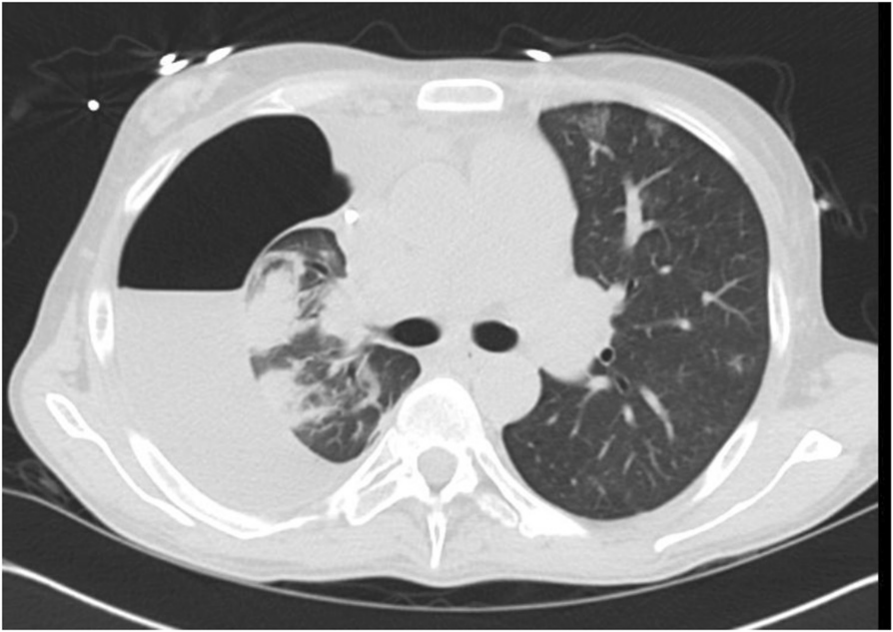
Chest CT post-OLT: Large right-sided hydropneumothorax with underlying visceral and parietal pleural thickening and fibrosis.

**Figure 3:**
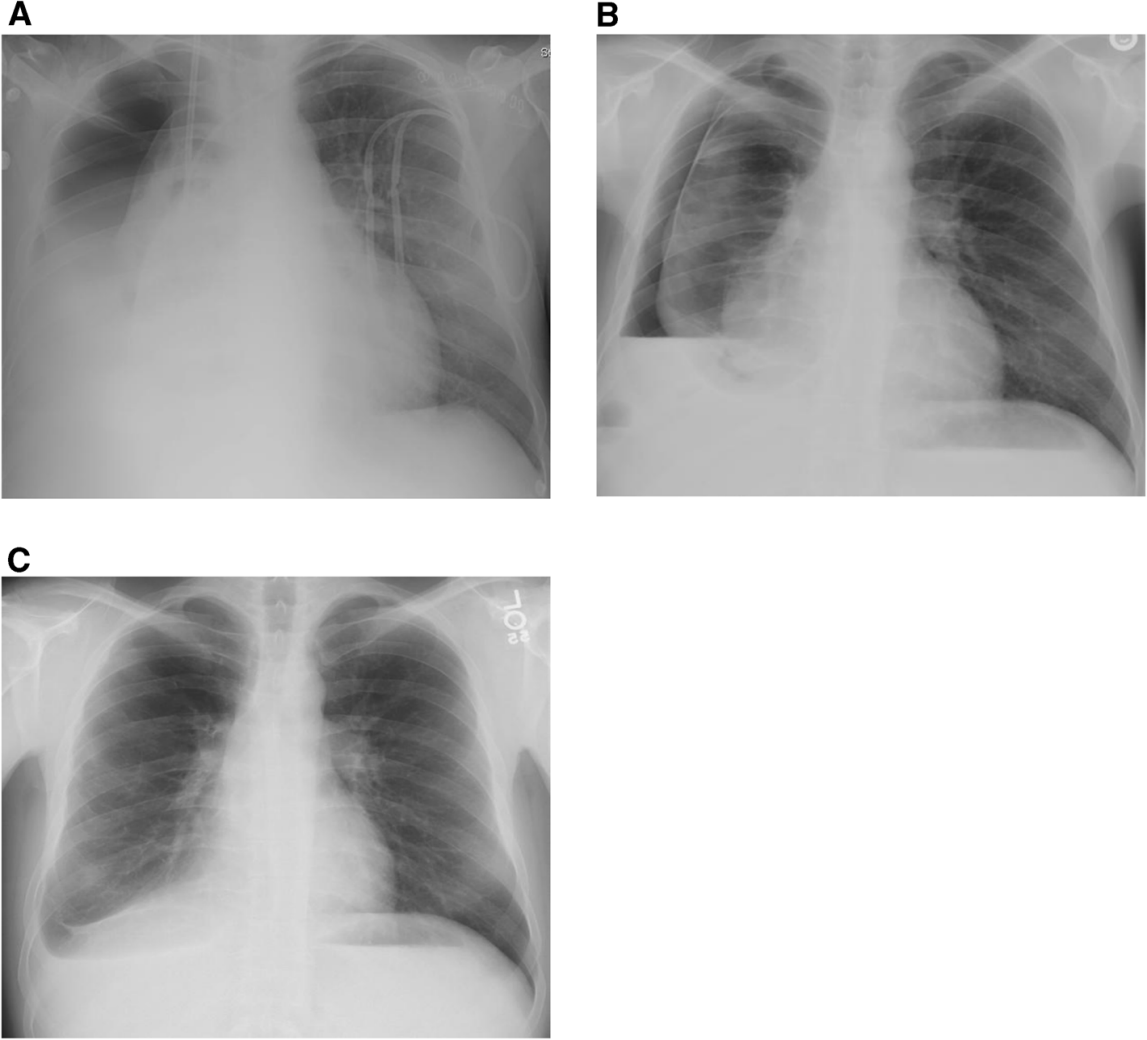
**Chest x-ray at discharge with a large right-sided hydropneumothorax (A).** Chest x-ray at 1 month after discharge with improvement in the right-sided hydropneumothorax (**B**). Chest x-ray at 6 months after discharge with significant right lung re-expansion with a small residual hydropneumothorax (**C**).

He subsequently underwent an uncomplicated OLT. A pre-operative chest x-ray revealed a large chronic right-sided pleural effusion. He was successfully extubated after OLT and doing well so no immediate intervention was performed on the effusion. On post-operative day (POD) #5, the patient developed respiratory distress with new left sided infiltrates consistent with pneumonia. He was started on antibiotics and a right-sided therapeutic thoracentesis was performed with removal of 1.3 liters of fluid. Post-thoracentesis chest imaging revealed a large hydropneumothorax suspicious for a pneumothorax ex-vacuo. A small-bore chest tube was inserted and placed on suction without any additional re-expansion of the lung or any change in his respiratory status (Figure 1). The chest tube was discontinued 2 days later and a subsequent chest computed tomography (CT) confirmed the presence of a large right-sided hydropneumothorax with underlying visceral and parietal pleural thickening and fibrosis causing restriction and constriction of the underlying right lung, consistent with “trapped lung” (Figure 2). Because there was no evidence of any endobronchial obstruction on CT we elected not to perform a diagnostic bronchoscopy to rule out endobronchial lesions. The patient’s respiratory status improved with antibiotics and no infection was identified in the pleural fluid cultures. Thoracic surgery was consulted for possible video-assisted thoracoscopic surgery (VATS); however, because the patient was doing well without any complaints of dyspnea and without hypoxemia, the decision was made to defer the surgery until his immunosuppression was tapered down in order to minimize wound healing complications. He was discharged home on POD #20 without need for supplemental oxygen. A repeat chest x-ray 1 month later showed improvement in the hydropneumothorax, and a chest x-ray obtained 6 months later showed near-complete re-expansion of his right lung without any surgical intervention (Figures 3A-C).

### Case 2

A 68-year old female with non-alcoholic steatohepatitis (NASH) complicated by a refractory right-sided hepatic hydrothorax (fluid to serum total protein ratio 0.3, fluid LDH < 2/3 upper limits of normal serum LDH) presented for an OLT evaluation. The diagnosis of NASH was made 14 years prior and for the 1 year prior to presentation to our hospital, she had required near weekly thoracenteses. The patient was subsequently admitted to our hospital with shortness of breath. Following a therapeutic thoracentesis, she was noted to have a pneumothorax. A 16 French chest tube was placed with no air leak or re-expansion of the lung following chest tube placement. A CT of the chest confirmed the presence of a hydropneumothorax without evidence of endobronchial obstruction. Thoracic surgery was consulted to consider her for definitive management of her pleural effusion because of the persistent high volume drainage (~700 cc per day) from the chest tube. The patient underwent VATS decortication and chemical pleurodesis with resolution of the hydropneumothorax. On POD #20 after her VATS, the patient underwent a successful OLT and did not suffer any further pulmonary or pleural complications.

### Case 3

A 74-year old male with cirrhosis secondary to hepatitis B complicated by a refractory right-sided hepatic hydrothorax (fluid to serum total protein ratio 0.2, fluid LDH < 2/3 upper limits of normal serum LDH) requiring near weekly thoracenteses for the preceding two years presented for an OLT evaluation. After a thoracentesis, he was found to have a pneumothorax and a small bore chest tube was placed by interventional radiology without subsequent re-expansion of the lung. A bronchoscopy was performed and did not reveal any endobronchial abnormalities. Given the complex loculated nature of the effusion, thoracic surgery recommended a thoracotomy with decortication. The operation was complicated by significant post-operative bleeding, and the patient was taken back to the operating room 3 days following the initial thoracotomy due to continued bleeding. Diffuse oozing was found intra-operatively from the parenchymal surface of the lung and the chest wall. The patient suffered a cardiac arrest and expired shortly after surgery.

### Case 4

**Figure 4:**
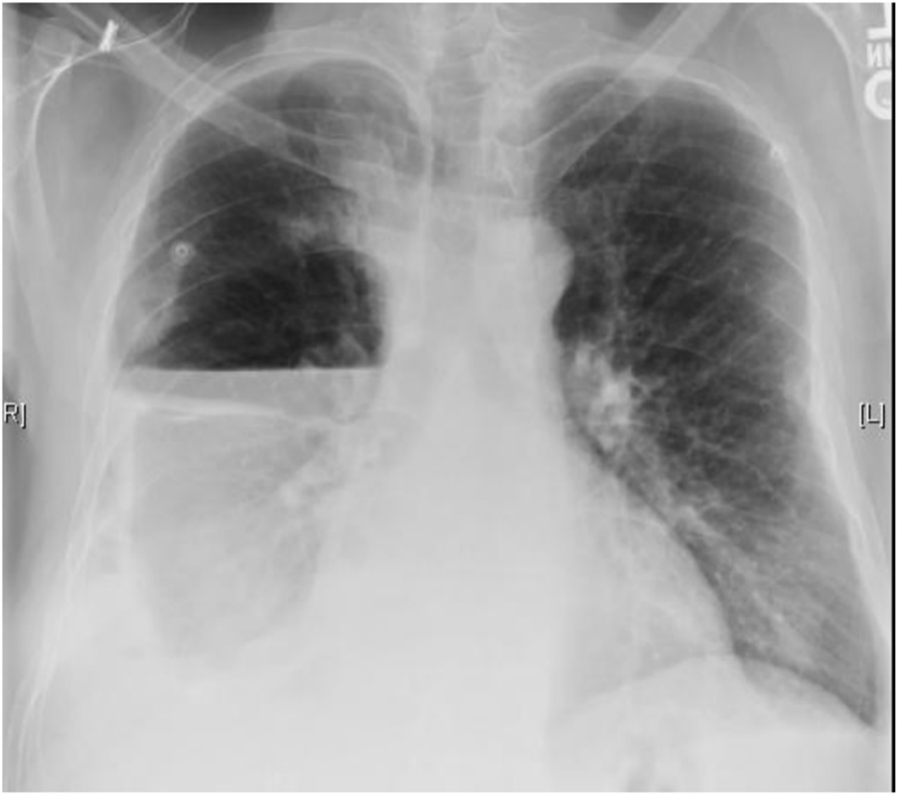
Chest x-ray after removal of indwelling pleural catheter showing a large right-sided loculated hydropneumothorax.

**Figure 5:**
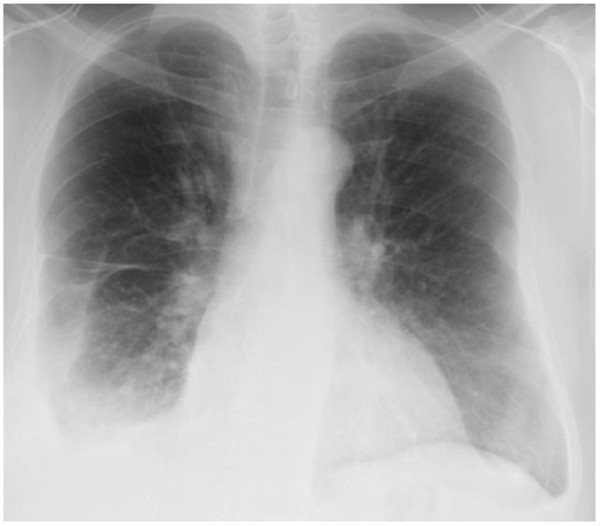
Chest x-ray at 1 month after discharge: Significant right lung re-expansion.

A 57-year old male with cirrhosis related to NASH diagnosed seven years prior, presented for an OLT evaluation. Prior to presentation, he had multiple chest tubes placed for a refractory right-sided hepatic hydrothorax (fluid to serum total protein ratio < 0.5, fluid LDH < 2/3 upper limits of normal serum LDH) with failed attempts at pleurodesis and had an indwelling pleural cavity drainage catheter placed at an outside hospital. Due to concerns for infection, the pleural catheter was later removed and he was managed with diuretics and frequent thoracenteses. Transjugular intrahepatic porto-systemic shunt (TIPS) was not initially felt to be an option secondary to a reported portal vein thrombosis. Approximately 3 months after the removal of the pleural catheter, another indwelling pleural catheter was placed by an outside hospital secondary to the patient’s difficulty with tolerating diuretics. He eventually underwent successful TIPS placement for the refractory hepatic hydrothorax, and the second indwelling pleural catheter was removed approximately 2 months after its placement. After removal of the second indwelling pleural catheter, chest x-ray revealed a large loculated hydropneumothorax (Figure 4), suspicious for trapped lung. Given that the patient remained asymptomatic without any oxygen requirement, the decision was made to defer any surgical intervention. He was discharged from the hospital and a follow-up chest x-ray one month later revealed significant improvement of his right-sided hydropneumothorax (Figure 5). He is currently doing well two years later and continues to await OLT.

## Conclusions

Historically, trapped lung was seen in conjunction with pneumothorax therapy used to treat tuberculosis [6]. Other conditions that have been associated with trapped lung include parapneumonic effusions, post-cardiac surgery, hemothorax, inflammatory pleuritis, chest radiation, and malignant pleural effusions [7]. The pathophysiology of pneumothorax ex-vacuo remains unclear. Several potential hypotheses have been proposed. The leading hypothesis involves the formation of a fibrous peel as the result of chronic inflammation that prevents the lung from re-expanding [8]. An alternative theory suggests that the depletion of surfactant, as a result of chronic atelectasis, is the reason for the lung to fail to re-expand [9]. In the setting of hepatic hydrothorax, we suspect a similar pathophysiology involving chronic inflammation and subsequent development of a fibrous layer along the visceral pleura. Table 1 summarizes the patient characteristics of this series. None of our patients had a documented infectious process in the pleural space at the time of the trapped lung diagnosis, and no endobronchial abnormalities were seen on chest CT or bronchoscopy (when performed). All patients did have a history of multiple thoracenteses and two patients had indwelling pleural cavity drainage catheters placed which could also have contributed to chronic inflammation and scarring along the pleura.

**Table 1:** Clinical characteristics of patients

***Case***	***1***	***2***	***3***	***4***
**Age (yrs)**	24	68	74	57
**Gender**	M	F	M	M
**Race/Ethnicity**	Hispanic	Hispanic	Caucasian	Middle Eastern
**Liver Disease**	Hepatitis C	NASH	Hepatitis B	NASH
OLT status*	Post	Pre	Pre	Pre
**Location (Right/Left)**	R	R	R	R
**Previous Indwelling Pleural Catheter (Yes/No)**	Y	N	N	Y (x 2)
**Infection in Pleural Fluid (Yes/No)**	N	N	N	N
**# Thoracenteses Performed**	~10	~5	>10	>15
**Chest Tube (Yes/No)**	Y	Y	Y	N
**Response**	N	N	N	N/A
**Surgical Intervention (Yes/No)**	N	Y	Y	N
**Response**	N/A	Y	N	N/A
**Cause of Death**	N/A	N/A	Bleeding after thoracic surgery	N/A

*at time of diagnosis of trapped lung.

Similar to experiences seen with trapped lung in malignant effusions, effective management of trapped lung in the presence of hepatic hydrothoraces remains extremely challenging [10]. Chest tube placement in this setting does not result in lung re-expansion, and surgical intervention can carry significant mortality and morbidity in patients with ESLD due to their underlying coagulopathy. Therefore, the ideal management for pneumothorax ex-vacuo in association with hepatic hydrothorax remains undefined. In our case series, chest tube placement at the initial diagnosis of trapped lung did not result in re-expansion of the lung in any of the patients. Two patients underwent surgical intervention: one patient had a successful outcome, while the other patient died from bleeding complications after thoracic surgery. The latter case underscores the high-risk nature of thoracic surgical intervention in this patient population. Finally, two patients subsequently had spontaneous near-resolution of their trapped lung without further thoracic interventions – one in the setting of immunosuppression after a liver transplant and one prior to liver transplant. The spontaneous improvements in the hydropneumothoraces suggest a potential for pleural remodeling and healing resulting in lung expansion over time, similar to that seen in some patients with trapped lung following an empyema.

To our knowledge, this is the first case series reported of the development of pneumothorax ex-vacuo in association with hepatic hydrothorax. Awareness of this phenomenon is essential in the care and management of patients with ESLD and recurrent hepatic hydrothoraces. In asymptomatic patients with pneumothoraces ex-vacuo in association with hepatic hydrothoraces, observation with conservative management may be appropriate prior to surgical interventions, especially in light of the bleeding complications that can occur with liver disease. It remains unclear whether the development of pneumothorax ex-vacuo would be preventable by keeping the pleural space as dry as possible to minimize chronic inflammation. Additional patients will need to be characterized and followed prospectively to make any further conclusions on the ideal management of this clinical dilemma.

## Consent

Written informed consent was obtained from the patient or next of kin for publication of this case report and any accompanying images. A copy of the written consent is available for review by the Series Editor of this journal.

## Abbreviations

CT: Computed tomography; ESLD: End-stage liver disease; NASH: Non-alcoholic steatohepatitis; OLT: Orthotopic liver transplantation; POD: Post-operative day; TIPS: Transjugular intrahepatic porto-systemic shunt; VATS: Video-assisted thoracoscopic surgery.

## Competing interests

We declare that there are no financial or non-financial competing interests associated with this manuscript.

## Authors’ contributions

YSK, TSW, IS, PE, MIS, AZ, and RB participated in the direct care of the patients reported in our series. YSK and TSW contributed to the case histories and the discussion portion of the manuscript. IS, MIS, and PE contributed to the discussion portion of the manuscript. AZ, RB, and CAL contributed to the revision and submission of the manuscript. All authors must read and approved the final manuscript.

## Authors’ information

YK fellow Pulmonary and Critical Care Program. IS Clinical Professor of Medicine. CL Assistant Clinical Professor, attending physician liver transplant intensive care unit. AZ fellow Liver Transplant Surgery. PE Assistant Clinical Professor, attending liver transplant intensive care unit. MIS Clinical Professor of Medicine. RB Professor, Chairman of UCLA Department of Surgery, Chief of UCLA Liver and Pancreas Transplantation Program, Director of the UCLA Liver Transplant Program. TW Assistant Clinical Professor, Director of Pulmonary Critical Care Consultation Service in the liver transplant intensive care unit.

## 

The work was performed at University of California Los Angeles Medical Center, Los Angeles, California.

## Pre-publication history

The pre-publication history for this paper can be accessed here:

http://www.biomedcentral.com/1471-2466/12/78/prepub
